# A stereo spatial decoupling network for medical image classification

**DOI:** 10.1007/s40747-023-01049-9

**Published:** 2023-04-17

**Authors:** Hongfeng You, Long Yu, Shengwei Tian, Weiwei Cai

**Affiliations:** 1 School of Information Science and Engineering, Xinjiang University, Urumqi, 830000 China; 2 Network Center, Xinjiang University, Urumqi, 830000 China; 3 Software College, Xinjiang University, Urumqi, 830000 China; 4 School of Artificial Intelligence and Computer Science, Jiangnan University, Wuxi, 214122 China

**Keywords:** Feature screening strategy, Multi-dimensional spatial attention, Neural networks

## Abstract

Deep convolutional neural network (CNN) has made great progress in medical image classification. However, it is difficult to establish effective spatial associations, and always extracts similar low-level features, resulting in redundancy of information. To solve these limitations, we propose a stereo spatial discoupling network (TSDNets), which can leverage the multi-dimensional spatial details of medical images. Then, we use an attention mechanism to progressively extract the most discriminative features from three directions: horizontal, vertical, and depth. Moreover, a cross feature screening strategy is used to divide the original feature maps into three levels: important, secondary and redundant. Specifically, we design a cross feature screening module (CFSM) and a semantic guided decoupling module (SGDM) to model multi-dimension spatial relationships, thereby enhancing the feature representation capabilities. The extensive experiments conducted on multiple open source baseline datasets demonstrate that our TSDNets outperforms previous state-of-the-art models.

## Introduction

**Fig. 1:**
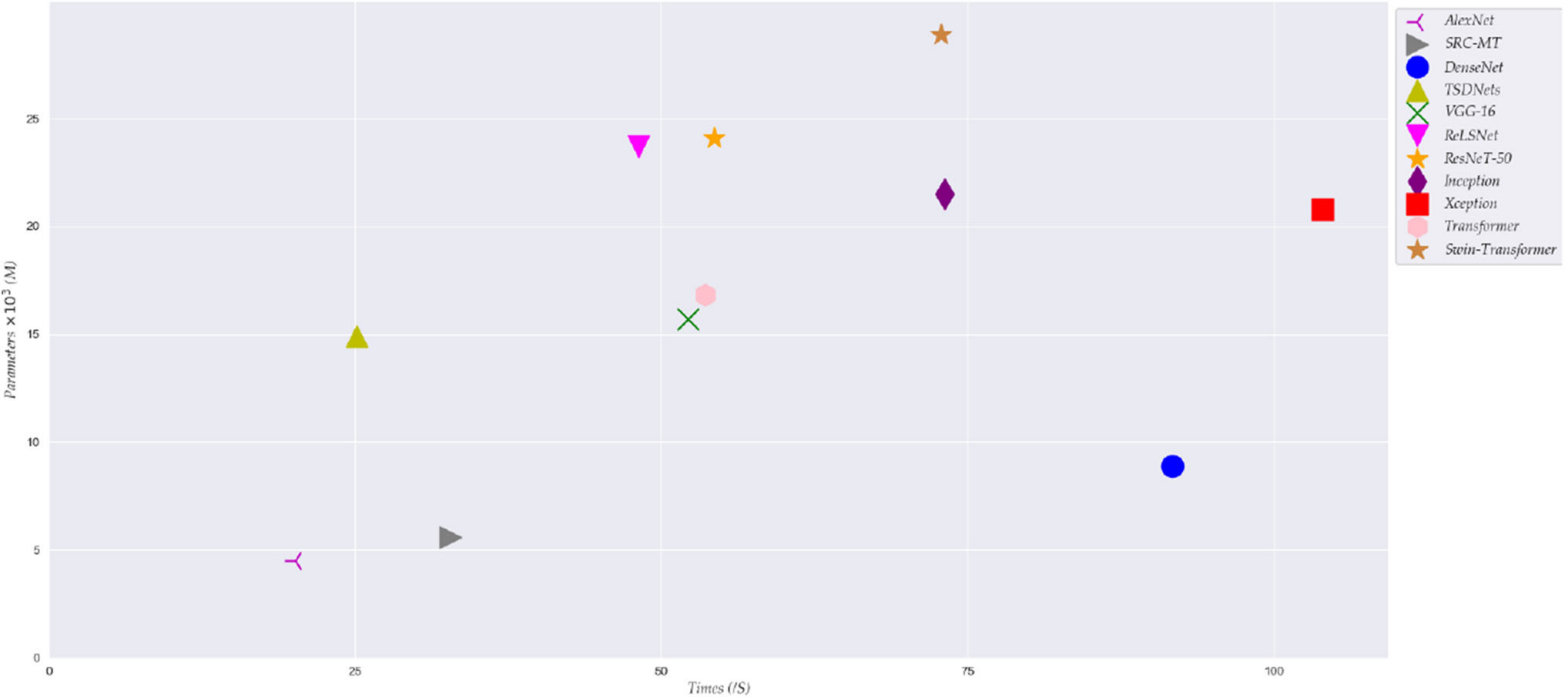
The parameters and running time of some representative models. The *y*-axis represents the number of parameters of a given model, while the *x*-axis represents the running time

Deep learning has always been the top priority of research in the field of medical image analysis, which effectively relieves the pressure of medical experts. In recent years, convolutional neural network (CNN) has been proposed and widely used in many real-world medical image analysis scenarios due its outstanding performance in classifying medical images, such as skin cancer image classification, and X-ray classification, etc. Recently, some variants of CNN model (e.g., DCNN, Resnet, Densenet, and multi-scale CNN [10, 18, 25]) have been attracting increasing attention to capture and exploit high-level discriminative images features. To better optimize the learning ability of the convolutional neural network, Khan M A et al. [9] fused the features of AlexNet and VGG16 in parallel and realized optimization at the same time to obtain the optimal features. To better detect the focus area of breast cancer, Irfan et al. [8] used DenseNet201 as the basic model, and fused 24 sets of convolutional feature vectors to obtain various semantic information of migration, and evaluated the proposed method through 10 fold cross validation. The accuracy of the proposed algorithm in breast cancer has reached 98. 9$\%$, which proves the feasibility of feature fusion. Although these methods have powerful feature representation capabilities, their efficiency and prediction accuracy are hindered by a large number of redundant features generated during model learning, while they are usually over-parameterized and computationally expensive. Figure 1 shows the parameters and running time of some representative models. Furthermore, attention mechanism has become an important research hotspot for medical image segmentation tasks. The concept of feature screening also plays a positive role in other fields [7, 29].

Many existing studies [26, 30] have shown that the attention mechanism can effectively enhance the representational ability of key features in the feature map by ignoring the irrelevant redundant information. However, for medical images, a specific disease object often appears in different imaging directions. When the pixel intensity of similar target objects changes slightly, it is difficult for the attention mechanism to distinguish the differences between pixels from a single direction. Therefore, it is necessary to conduct effective exploration from different directions. Some studies [4, 6, 16] have shown that use attention to automatically extract the appropriate feature from two directions, thereby better capturing the dependencies between features. However, they ignore the problem of redundant features, which may cause the model to fail to learn useful information, increasing the difficulty of model learning.

In previous studies [21], the shallow features are directly introduced into the deep features. Although the introduction of shallow features can bring more semantic information to the deep features, the introduction of too much information with weak correlation tends to reduce the quality of the deep feature maps. Therefore, reasonable selection of feature points can not only improve the sparsity of feature maps, but also avoid feature reuse and improve model efficiency.

In this paper, we propose a stereo spatial decoupling network (TSDNets) for medical image classification that utilizes three sets of attention mechanisms to assign three sets of weights to each feature point. Moreover, we adopt a feature screening strategy to suppress redundant features and build gating strategies to improve the quality of features.

- We propose a stereo spatial decoupling network (TSDNets) to explore the spatial guidance relationship of the object from three directions of horizontal, vertical, and depth of the medical image. Specifically, the attention in this paper is more accurate than the traditional one-way attention mechanism screening features from multiple perspectives. At the same time, the attention mechanism in this paper plays a role in filtering features, and it is not a feature fusion directly, so compared with the traditional attention mechanism [27, 33], the parameter operation is less.
- We developed a cross-feature screening module (CFSM) that uses a two-gate threshold screening strategy to generate three types of features, namely important features, secondary features, and redundant features, and targets them for deep feature fusion.
- We constructs a semantic guided decoupling module (SGDM), which implements feature selection by setting different gate thresholds for shallow features and deep features respectively, thereby extracting more discriminative features.

## Related work

In recent years, deep learning has received significant research attention from academia and industry. A widely used method in medical image classification is the convolution neural network (CNN) [2, 12]. However, traditional medical image datasets are usually small and complex, especially when there are specific targets or specific regions in the image that are similar to the surrounding background. Directly applying traditional CNN to medical image classification may be difficult to establish effective spatial associations between features or similar images. To improve the feature representation, various solutions [4, 6, 21] have recently been proposed to force CNN to focus on specific regions, and rely on domain knowledge to obtain information [12, 20]. For example, in [17, 32], authors obtain important relevant area information according to the gray value of the medical image. However, in most cases, domain knowledge is limited (only suitable for specific tasks). Moreover, to describe the targets from multiple levels with rich feature representations, [12, 20] designed a multi-scale feature extractor to describe the classification objects from multiple aspects using different scale convolution kernels, and then used a simple linear fusion method to represent the features. Although these methods demonstrate the effectiveness of multi-scale features in medical image classification, the extracted redundant information can easily reduce the expression of key features when multi-scale information flows are transferred between layers.

To reduce irrelevant redundant information, the attention mechanism has been widely used in medical image classification, which can suppress redundant features through the weights generated by attention mechanism. There have been several attempts to improve the feature representation ability of model using local or global based attention mechanism [6, 13, 26, 30, 35], which allows the network to focus on the most key features or areas, thereby suppressing redundant features. Moreover, the disease regions in a medical image are similar to the surrounding background, which makes it difficult to describe the key regions by a single-direction attention mechanism and establish effective spatial relationships. To address this limitation, various attention-based methods [6, 16] have been proposed to locate key features to better establish the relationship between features. Although these methods [24, 28] can improve the quality of feature maps, they ignore the relationship between spatial semantic information and feature map semantic information, and lack in-depth analysis of redundant information. In this paper, we reconstruct the connection between redundant features to assist important semantic establishment and enhance spatial semantic information.

In medical image analysis, introducing shallow features into high-order feature maps is an important fusion method, where shallow features can assist higher-order features to capture finer semantic information. However, most of existing studies ignore the influence of redundant features on key features, which weakens the representation ability of key features. Feature selection methods [15, 19] have been widely used in many areas, which can be used to improve the model performance effectively. Some simple linear or filtering methods can also be used to filter features [3, 11, 15], such as point mutual information (PMI), PCA, Gaussian filtering, etc. These methods are mainly applied in the image pre-processing. To solve this limitation, Dropout [31, 34] and regularization techniques have been used to prevent the neural network from falling into a local optimum. Moreover, although the attention mechanisms can suppress the redundant features in the feature selection process, most attention based methods do not directly discard these feature points, which weakens the representation ability of key features. In this paper, we propose a spatial decoupling and cross-feature screening strategy based feature selection scheme to minimize the influence of redundant information on key features.

## TSDNets

**Fig. 2:**
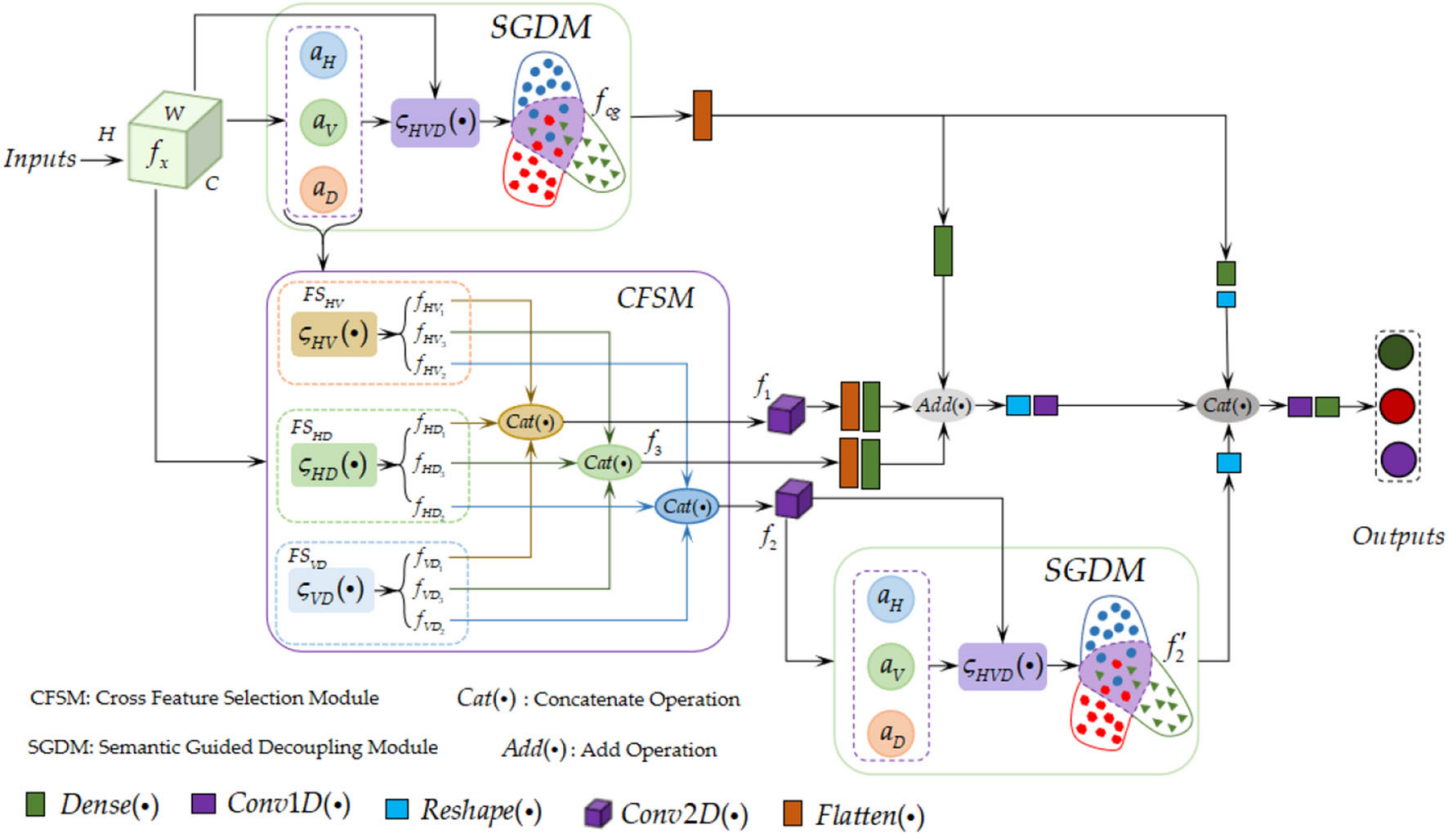
The overall architecture of the proposed TSDNets. $a_H, a_V, a_D$ represent the horizontal weight matrix, vertical weight matrix and depth weight matrix, respectively; $\varsigma _{H V D}(\bullet )$ is the intersection threshold filter function; $\zeta _{H V}(\bullet ), \varsigma _{H D}(\bullet ), \varsigma _{V D}(\bullet )$ represent the cross filtering function of horizontal and vertical, the cross filtering function of horizontal and depth, the cross filtering function of vertical and depth directions, respectively; $f_{cg}$ represents the optimal low-level global semantic feature; $f'_{cg}$ represents the optimal high-order local semantic feature; $f_{x} \in R^{H \times W \times C}$ is the initial features in the input layer, where *H*, *W*, *C* represent the height, width, and channel respectively

The proposed TSDNets primarily consists of a cross-feature screening module (CFSM) and a semantic guided decoupling module (SGDM). The TSDNets first captures the shallow features of the objects by the convolutional operations, and then uses three different attention mechanisms to decompose the shallow features into three corresponding weight matrices $a_H$, $a_V$, and $a_D$ from the horizontal, vertical, and depth directions. Three new matrices of $a_{HV}$, $a_{HD}$, and $a_{VD}$ are generated by combining the three weight matrices of $a_H$, $a_V$, and $a_D$ in pairs. Based on the dual-gating threshold, CFSM divides each of weight matrices, $a_{HV}$, $a_{HD}$, and $a_{VD}$, into three levels: important, secondary and redundant. Then, three new feature matrices ($f_1$, $f_2$, and $f_3$) are obtained by fusing the feature information of the corresponding levels. Meanwhile, the SGDM extracts the optimal shallow global semantic features $f_{cg}$ of $f_1$, as well as the optimal deep local semantic features $f'_{cg}$ of the secondary features $f_2$. Considering that redundant features only contain less useful semantic information. We compress the redundant features into a one-dimensional dense vector to reconstruct the relationship between them. The overall flow of the proposed algorithm is shown in Fig. 2.

In this part, we propose a stereo decoupling attention mechanism, which can extract the key features by assigning weights to each feature, and decompose shallow features from three directions of horizontal, vertical, and depth. As a result, we can obtain the horizontal attention weight matrix $a_H$, the vertical attention weight matrix $a_V$, and the depth attention weight matrix $a_D$. The horizontal attention weight matrix $a_H$ can be described by

1 $$\begin{aligned} {\left\{ \begin{array}{ll} \displaystyle a_{i}=\sum _{i=1}^{n} \frac{\exp \left( e_{i, j}\right) }{\sum _{k=1}^{n} \exp \left( e_{i k}\right) } h_{j} \\ \displaystyle a_{H}=\left\{ a_{1}, \ldots , a_{i-1}, a_{i}\right\} \end{array}\right. } \end{aligned}$$

where $e_{i,j}$ is the weight coefficient assigned by the attention mechanism; $h_j$ represents the hidden layer; $a_i$ represents the horizontal attention weight coefficient of the *i*-th feature. Similarly, we can get $a_V$ and $a_D$.

### Cross feature screening module (CFSM)

This cross-feature screening module (CFSM) is mainly divided into two parts: the dual gate threshold screening and the feature aggregation. In the dual gate threshold screening, the above three weight matrix $a_H$, $a_V$, and $a_D$ are divided into three levels: important, secondary, and redundant, according to two thresholds $T_1$ and $T_2$. After that, we can obtain three new weight matrices $a_{HV}$, $a_{HD}$, and $a_{VD}$ by reconstructing $a_H$, $a_V$, and $a_D$ in pairs. Then, we average the three weight matrices $a_{HV}$, $a_{HD}$, and $a_{VD}$ to obtain three mean matrices $\frac{a_{HV}+a_{HD}}{2}$, $\frac{a_{HD}+a_{VD}}{2}$, and $\frac{a_{VD}+a_{HV}}{2}$, where each weight value in the three mean matrices is compared to $T_1$ and $T_2$. When the feature points are graded, they are more stable if the mean of any two groups of weights is calculated. If the value is greater than $T_1$, it means that the feature *i* contains the important semantic information. Analogously, if the value is less than $T_2$, it means that the feature *i* only contains redundant information. Next, we divide the weight matrix $a_{H,V}$ into three 0-1 matrices (where 0 represents eliminating the feature *i* and 1 represents retaining the feature *i*), namely important matrix ${\hat{a}}_{H V_{1}}$, secondary matrix ${\hat{a}}_{H V_{2}}$ and redundant matrix ${\hat{a}}_{HV_{3}}$.

2 $$\begin{aligned} {\left\{ \begin{array}{ll} \displaystyle {\widehat{a}}_{H V_{1}}^{i}=\left( \varsigma _{H V}^{i}(1), \frac{a_{HV}+a_{HD}}{2}>T_{1} \Vert \varsigma _{H V}^{i}(0), \frac{a_{HV}+a_{HD}}{2}<T_{1}\right) \\ \displaystyle {\widehat{a}}_{H V_{2}}^{i}=\left( \varsigma _{H V}^{i}(1), T_{2}<\frac{a_{HV}+a_{HD}}{2}<T_{1} \Vert \varsigma _{H V}^{i}(0), \right. \\ \displaystyle \qquad \qquad \left. \left\{ \frac{a_{HV}+a_{HD}}{2}<T_2, T_1<\frac{a_{HV}+a_{HD}}{2} \right\} \right) \\ \displaystyle {\widehat{a}}_{H V_{3}}^{i}= \left( \varsigma _{H V}^{i}(0), \frac{a_{HV}+a_{HD}}{2}>T_{2} \Vert \varsigma _{H V}^{i}(1), \frac{a_{HV}+a_{HD}}{2}<T_{2}\right) \end{array}\right. } \end{aligned}$$

Analogously, we can obtain the corresponding reconstructed 0-1 matrices, ${{\widehat{a}}_{HD_1}}$, ${{\widehat{a}}_{HD_2}}$, ${{\widehat{a}}_{HD_3}}$, ${{\widehat{a}}_{VD_1}}$, ${{\widehat{a}}_{VD_2}}$, ${{\widehat{a}}_{VD_3}}$. Then, we multiply the feature matrix with three sets of 0-1 matrices to get a new three sets of feature matrices, $f_{H V_{1}}, f_{H V_{2}}$, $f_{H V_{3}}$.

3 $$\begin{aligned} {\left\{ \begin{array}{ll} f_{H,V_{1}}=\varphi ({\widehat{a}}_{H, V_{1}} \cdot f_{x})\\ f_{H, V_{2}}=\varphi ({\widehat{a}}_{H,V_{2}} \cdot f_{x}) \\ f_{H,V_{3}}=\varphi ({\widehat{a}}_{H,V_{3}} \cdot f_{x}) \end{array}\right. } \end{aligned}$$

For $$a_{HD}$$ and $$a_{VD}$$, we can obtain the corresponding feature matrices $$f_{H D_{1}},$$ $$ f_{H D_{2}},$$ $$ f_{H D_{3}},$$ $$ f_{V D_{1}},$$ $$ f_{V D_{2}}$$, $$f_{V D_{3}}$$. Next, we compute the middle-level feature $$f_1$$, $$f_2$$, $$f_3$$ as follows.

4 $$\begin{aligned} {\left\{ \begin{array}{ll} f_{1}={\text {Cat}}(f_{H V_{1}}, f_{H D_{1}}, f_{V D_{1}}) \\ f_{2}={\text {Cat}}(f_{H V_{2}}, f_{H D_{2}}, f_{VD_{2}}) \\ f_{3}={\text {Cat}}(f_{H V_{3}}, f_{H D_{3}}, f_{V D_{3}}) \end{array}\right. } \end{aligned}$$

where ${\text {Cat}}$ represents the concatenate operation.

### Semantic guided decoupling module(SGDM)

To improve the quality of features, we develop a semantic guided decoupling module (SGDM), which introduce high-quality shallow features in deep feature fusion. By taking the square root of the sum of the squares of the weight matrices in each direction ($a_H$, $a_V$ and $a_D$), we can get two new sets of shallow global semantic feature’s weight matrices ${S}_{H V D}$ and deep local semantic feature’s weight matrices ${D}_{H V D}$. The purpose is that when the weight of any direction of the feature point is greater than the set threshold, it has stronger discriminability, so that the model retains richer semantic information.

5 $$\begin{aligned} {\left\{ \begin{array}{ll} S_{H V D}=\sqrt{(a_{H})^{2}+(a_{V})^{2}+(a_{D})^{2}}\\ D_{H V D}=\sqrt{(a_{H})^{2}+(a_{V})^{2}+(a_{D})^{2}} \end{array}\right. } \end{aligned}$$

Then, SGDM performs feature screening for the shallow global features $f_x$ and deep local features $f_y$. For the shallow global features $f_{x}$ (as shown in Fig. 2), we compare the weight value in $S_{H V D}$ with the threshold $T_1$ to generate the corresponding shallow global 0–1 matrix ${\widehat{S}}_{H V D}$. Similarly, for deep local features $f_{y }$ (as shown in Fig. 2), the corresponding deep local 0–1 matrix ${\widehat{D}}_{H V D}$ can be generated by comparing the $D_{H V D}$ with the threshold $T_3$. Specifically, the feature screening is the same as in Sect. “Cross feature screening module (CFSM)”, which can be described as follows.

6 $$\begin{aligned} {\left\{ \begin{array}{ll} {\widehat{S}}_{H V D}^{i} =(S_{H V D}^{i}(1), S_{H V D}>T_{1} \Vert S_{H V D}^{i}(0), S_{H V D}<T_{1}) \\ {\widehat{D}}^{i}_{H V D} =(D_{H V D}^{i}(1), D_{H V D}>T_{3} \Vert D_{H V D}^{i}(0), D_{H V D}<T_{3}) \end{array}\right. } \end{aligned}$$

Finally, a set of optimal shallow global semantic features $f_{cg}$ and a set of optimal deep local semantic features $f'_{cg}$ are generated.

7 $$\begin{aligned} {\left\{ \begin{array}{ll} f_{c g}=\varphi ({\widehat{S}}_{H V D} \cdot f_{x}) \\ f_{c g}'=\varphi ({\widehat{D}}_{H V D} \cdot f_{y}) \end{array}\right. } \end{aligned}$$

where $\varphi ()$ represents the matrix multiplication operation.

### Feature fusion

Feature fusion can improve the accuracy of medical image classification. To improve the feature representation ability of redundant features $f_{3}$, we project the two-dimensional features $f_{3}$ into a one-dimensional vector space to reconstruct the feature relationships. Then, the shallow global features and important features are fused to obtain fused features $f_{m}$. This process can be described as follows:

8 $$\begin{aligned} {\left\{ \begin{array}{ll} f_{3}'={\text {Flatten}}({\text {Dense}}(f_{3})) \\ f_{1}'={\text {Flatten}}({\text {Dense}}({\text {Conv2D}} (f_{1}))) \\ f_{m}={\text {Add}}(f_{3}', f_{1}', {\text {Dense}}(\text {Flatten}(f_{c g})) \end{array}\right. } \end{aligned}$$

After that, we fuse $f_{c g}$, $f_{m}$ and the high-order features $f_{c g}^{\prime }$ to obtain the deep features. This process can be described as follows:

9 $$\begin{aligned} {\left\{ \begin{array}{ll} f_{c g}^{\prime \prime }={\text {Reshape}}({\text {Dense}}({\text {Flatten}}(f_{c g}))) \\ f_{m}^{\prime }={\text {Conv1D}} ({\text {Reshepe}}(f_{m})) \\ {\hat{f}}_{c g}={\text {Reshape}}(f_{c g}^{\prime }) \\ f_{i}={\text {Cat}}(f_{c g^{\prime }}^{\prime \prime }, f_{m}^{\prime }, {\hat{f}}_{c g}) \end{array}\right. } \end{aligned}$$

Next, we can get the final output feature *O*, as follows.

10 $$\begin{aligned} O={\text {Dense}}({\text {Conv1 D}} (f_{i})) \end{aligned}$$

Finally, the SoftMax classifier is used to output the classification probability.

## Feature visualization for CFSM and SGDM

**Fig. 3:**
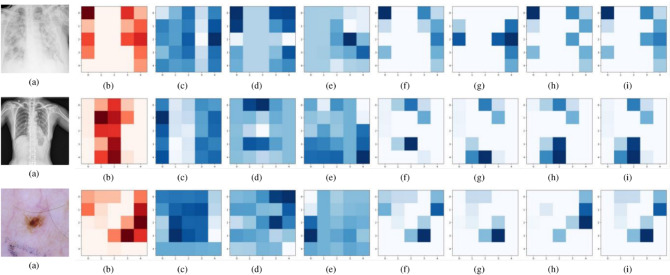
The weighting process of different attention mechanisms. The first row is the visualization of COVID deep feature weights; the second row is the visualization of CXR deep feature weights; the third row is the visualization of ISIC deep feature weights

In this part, we validates the correctness on theoretical deduction as well as obtains some important conclusions by case study and visualization analysis. Figure 3 shows the visual analysis results of 5 $\times$ 5 deep features with their weight coefficients, where color denotes to the weight of the attention matrix. The red cell indicates that the feature point contains more semantic information, and the blue cell indicates that the feature point has a greater weight. Figure 3a is the original image. Figure 3b represents the deep feature; Fig. 3c–e represent the weight coefficients generated by vertical attention, horizontal attention, and deep attention, respectively; Fig. 3f represents the weight coefficients generated by the horizontal attention and vertical attention in CFSM module; Fig. 3g represents the weight coefficients generated by the horizontal attention and the depth attention in CFSM module; Fig. 3h represents the weight coefficients generated by the vertical attention and the depth attention in CFSM module; Fig. 3i represents the weight coefficients generated by these three attention mechanisms in the SGDM module.

As can be seen from Fig. 3c–e, the weight visualization results show that if using only one attention mechanism, the effect of weight assignment performs the worst. As shown in Fig. 3f–h, when we further use two attention mechanisms in CFSM module, this obviously produces information gain. From Fig. 3i, we use three attention mechanisms in SGDM module, the generated features contain more semantic information. This shows the proposed TSDNets is effective at guiding the attention weights to select the useful features.

## Experiments and results

### Datasets

We verify the MAS-Net on three datasets which are briefly described in the following paragraphs.

*Shenzhen dataset—Chest X-ray database* [5]. This dataset was constructed by the National Library of Medicine of Maryland, USA, and the Third People’s Hospital of Shenzhen, China. This dataset consists of 662 chest X-rays images, including 336 tuberculosis disease images (TB) and 326 normal medical images (Nor). The size of each image is 3000 $\times$ 2900 $\sim$ 3000.

*COVID-19 radiography database* [1]. This dataset was jointly produced by researchers from Qatar University and Dhaka University. There are only 219 COVID-19 images in the original dataset. To balance this dataset, the number of images reached 1200 through post-supplementation. The data consist of three types of medical chest X-ray images: viral pneumonia (VP, 1345 images), normal (Nor, 1341 images), and COVID (COVID, 1200 images). The size of each image is 1024 $\times$ 1024.

*ISIC2018 dataset * [22]. This dataset is the largest public dataset of skin diseases, consisting of 10,015 skin disease images. Different types of skin disease images in this dataset were acquired using different types of imaging equipments. The dataset contains 7 types of skin diseases: E MELANOMA (MEL, 1113), MELANOCYTIC NEVUS (NV, 6705), BASAL CELL CARCINOMA (BCC, 514), ACTINIC KERATOSIS (AKIEC, 327), BENIGN KERATOSIS (BKL, 1099), DERMATO FIBROMA (DF, 115) and VASCULAR LESIONS (VASC, 142). The size of each image is 600 $\times$ 450.

For training and testing purposes for our model, we have our data broken down into three distinct datasets with a ratio of 7:1:2, i.e, the training set, the validation set and the test set. All medical images are compressed to 256 $\times$ 256. We train our model using the deep learning framework Keras on the desktop computer (Tesla V100 with 16 G of RAM). We set the learning rate as 0.0001, and use Adam as the optimizer.

### Evaluation metrics

We evaluate each model and perform various ablation experiments using the average accuracy (AA), overall accuracy (OA) and Kappa coefficient (Kappa) metrics.

11 $$\begin{aligned} \text {AA}&=\frac{1}{S}\left( \frac{n_{1}}{m_{1}}+\frac{n_{2}}{m_{2}}+\cdots +\frac{n_{s}}{m_{s}}\right) \end{aligned}$$

12 $$\begin{aligned} \text {OA}&=\frac{n_{1}+n_{2}+\ldots +n_{s}}{m_{1}+m_{2}+\cdots +m_{s}}\end{aligned}$$

13 $$\begin{aligned} \text{ Kappa }&=\frac{OA-\frac{\left( n_{1} \times m_{1}+n_{2} \times m_{2}+\cdots +n_{s} \times m_{s}\right) }{S \times S}}{1--\frac{\left( n_{1} \times m_{1}+n_{2} \times m_{2}+\cdots +n_{s} \times m_{s}\right) }{S \times S}} \end{aligned}$$

where $n_i$ represents the number of samples belonging to the *i*th class, which are perfectly classified; $m_i$ represents the total number of *i*th class; and *S* represents the number of classes.

### Comparison to other state-of-the-art methods

For fair comparison, in this experiment, we compare the proposed TSDNets classification framework with other state-of-the-art medical image classification models. Table 1 lists the experimental results on different data sets. We can see that the proposed TSDNets achieves the best performance on the three benchmark datasets. Compared with SRC-MT, the Kappa value on the three datasets is increased by 4.54%, 0.02% and 2.13%, respectively. This may be because the proposed TSDNets can enrich the shallow global semantics of the target objects by the semantic guided decoupling module (SGDM), while providing the effective prior information for feature extraction. Moreover, CFSM can capture more favorable local features, making the network pay more attention to subtle changes in the objects. Overall, the proposed TSDNets method is effective for medical image classification.

**Table 1:** Experimental results of different classification models

Model	ChinaSet			COVID-19			ISIC			ChinaSet	
	OA	AA	Kappa	OA	AA	Kappa	OA	AA	Kappa	Parameters	FLOPs
DenseNet-121	0.8863	0.8902	0.7730	0.9818	0.9822	0.9726	0.7540	0.6008	0.5059	7.2 M	14.9 M
ResNet-50	0.8566	0.8655	0.7122	0.9870	0.9874	0.9804	0.7605	0.6190	0.4929	23.5 M	47.4 M
VGG-16	0.8712	0.8730	0.7426	0.9779	0.9783	0.9668	0.7202	0.5146	0.3863	14.8 M	29.6 M
Xception	0.8787	0.8870	0.7580	0.9844	0.9847	0.9765	0.7404	0.5044	0.4424	21.2 M	42.8 M
InceptionV3	0.8712	0.8750	0.7427	0.9831	0.9837	0.9746	0.7852	0.6053	0.5670	22.0 M	44.2 M
AlexNet	0.8787	0.8870	0.7580	0.9792	0.9795	0.9687	0.7973	0.6533	0.5835	4.3 M	8.7 M
ReLSNet	0.8787	0.8870	0.7580	0.9870	0.9874	0.9804	0.7888	0.6418	0.5878	18.6 M	37.1 M
SRC-MT	0.8939	0.8892	0.7882	0.9870	0.9874	0.9804	0.7958	0.6795	0.5792	25.9 M	52.1 M
Transformer	0.8712	0.8750	0.7427	0.9870	0.9874	0.9804	0.7864	0.6506	0.5824	16.8 M	33.7 M
Swin-Transformer	0.8787	0.8870	0.7580	0.9883	0.9889	0.9824	0.8012	0.6931	0.5903	28.9 M	57.4 M
TSDNets	0.9167	0.9238	0.8336	0.9883	0.9889	0.9824	0.8044	0.7144	0.6005	32.3 M	65.2 M

Due to the large number of categories in ISIC and the small differences between the target categories, all the baseline methods have achieved poor classification performance on the ISIC dataset. For example, the kappa values of AlexNet and ReLSNet are only 0.5835 and 0.5878, which are 1.7% and 1.26% lower than TSDNets, respectively. Transformer [14] Tand Swin Transformer [23] Tdo not work well in small data ChinaSet, but their effects are significantly improved when the number of data set samples is increased. Our proposed model TSDNets is better applicable to all data sets.

**Fig. 4:**
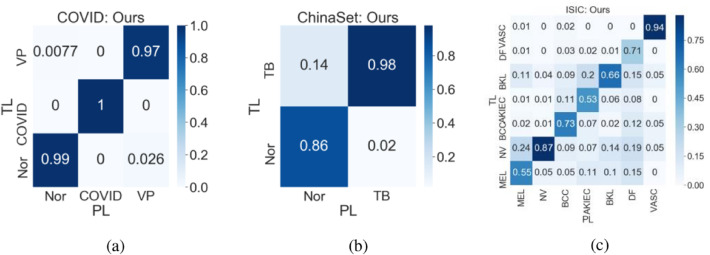
The confusion matrix of TSDNets on the three datasets, COVID-19, ChinaSet, and ISIC. “TL” represent the group truth labels; “PL” represent the predictions

When the category and data size are small (i.e., the ChinaSet dataset has 662 images in 2 categories), the proposed TSDNets gives the best performance under all metrics. This further demonstrates that TSDNets has good robustness and generalization. Specifically, Fig. 4 shows the confusion matrix of TSDNets on the three datasets.

### Ablation study

#### Effects of features

In the experiment, the selection of features has an important influence on the experimental results. We verified the impact of the different features $f_{cg}$, $f_1$, $f_2$, $f_3$ (see Section III for details) on the classification performance, as shown in Table 2. We can see that the $Model\_nof_{cg}$) is closest to our method in all metrics on the three benchmark datasets. The performance of $Model\_nof_{cg}$ in the Kappa is 9.08%, 3.02%, and 7.56% higher than the other three methods $Model\_nof_{1}$, $Model\_nof_{2}$, and $Model\_nof_{3}$, respectively. This shows that the shallow global features $f_{cg}$ have smaller advantages compared to other features. We observe that the performance of $Model\_nof_{3}$ was worse than that of other models. This shows that although the redundant feature $f_{3}$ contains only a small amount of useful semantic information. Moreover, this also demonstrates that redundant features in a specific dimensional space could improve the classification performance.

**Table 2:** Experimental results of different features comparisons

Dataset	Model	OA	AA	Kappa
COVID-19	$Model\_nof_{cg}$	0.9850	0.9844	0.9766
	$Model\_nof_{1}$	0.9825	0.9818	0.9727
	$Model\_nof_{2}$	0.9810	0.9805	0.9707
	$Model\_nof_{3}$	0.9733	0.9727	0.9590
	TSDNets	0.9889	0.9883	0.9824
ChinaSet	$Model\_nof_{cg}$	0.9084	0.9015	0.8034
	$Model\_nof_{1}$	0.8623	0.8560	0.7126
	$Model\_nof_{2}$	0.8966	0.8864	0.7732
	$Model\_nof_{3}$	0.8715	0.8636	0.7278
	TSDNets	0.9238	0.9167	0.8336
ISIC	$Model\_nof_{cg}$	0.7962	0.6850	0.5927
	$Model\_nof_{1}$	0.7903	0.6605	0.5641
	$Model\_nof_{2}$	0.7913	0.6492	0.5833
	$Model\_nof_{3}$	0.7686	0.5553	0.5314
	TSDNets	0.8044	0.7144	0.6006

**Table 3:** Experimental results of different gated thresholds comparisons. $T_1$, $T_2$, and $T_3$ indicate different gated thresholds respectively

Dataset	Model	OA	AA	Kappa
COVID-19	$T_1=0.5$, $T_2=0.3$, $T_3=0.5$	0.9876	0.9870	0.9805
	$T_1=0.6$, $T_2=0.2$, $T_3=0.5$	0.9813	0.9805	0.9707
	$T_1=0.6$, $T_2=0.2$, $T_3=0.4$	0.9863	0.9857	0.9785
	$T_1=0.6$, $T_2=0.3$, $T_3=0.6$	0.9848	0.9844	0.9766
	$T_1=0.6$, $T_2=0.4$, $T_3=0.5$	0.9850	0.9844	0.9766
	$T_1=0.7$, $T_2=0.5$, $T_3=0.5$	0.9812	0.9805	0.9707
ChinaSet	$T_1=0.5$, $T_2=0.3$, $T_3=0.5$	0.8931	0.8864	0.7731
	$T_1=0.6$, $T_2=0.2$, $T_3=0.5$	0.8804	0.8788	0.7573
	$T_1=0.6$, $T_2=0.2$, $T_3=0.4$	0.8968	0.8939	0.7881
	$T_1=0.6$, $T_2=0.3$, $T_3=0.6$	0.8968	0.8939	0.7881
	$T_1=0.6$, $T_2=0.4$, $T_3=0.5$	0.8903	0.8864	0.7730
	$T_1=0.7$, $T_2=0.5$, $T_3=0.5$	0.8799	0.8787	0.7577
ISIC	$T_1=0.5$, $T_2=0.3$, $T_3=0.5$	0.6174	0.7757	0.5371
	$T_1=0.6$, $T_2=0.2$, $T_3=0.5$	0.6134	0.7767	0.5563
	$T_1=0.6$, $T_2=0.2$, $T_3=0.4$	0.6398	0.8034	0.5883
	$T_1=0.6$, $T_2=0.3$, $T_3=0.6$	0.6533	0.8120	0.5933
	$T_1=0.6$, $T_2=0.4$, $T_3=0.5$	0.6189	0.7857	0.5703
	$T_1=0.7$, $T_2=0.5$, $T_3=0.5$	0.6573	0.7868	0.5512

#### Effects of gated thresholds

To verify the effectiveness of the gated threshold screening strategy, we conducted a large number of experiments. Table 3 shows the results of the proposed TSDNets with different gated thresholds. In the COVID-19 dataset, when $T_2$ and $T_3$ are fixed, as the gated threshold $T_1$ increases, the classification performance increases, which then decreases gradually. With the increase of $T_2$, the classification performance shows the same trend when $T_1$ and $T_3$ are fixed. The reason may be that when the gating threshold is low, irrelevant redundant information cannot be effectively filtered, thereby reducing the overall classification performance of the model. Moreover, when the gating threshold increases to a certain peak value, it can effectively filter irrelevant redundant information while preserving spatial semantic details. However, when the gating threshold continues to increase, some useful features may be filtered out, resulting in insufficient feature representation. From Table 3, we can see that when $T_1=0.5$, $T_2=0.3$, $T_3=0.5$, the proposed TSDNets achieves the best performance.

## Conclusion

This paper proposes a stereo spatial decoupling network (TSDNets) for medical image classification. We use the semantic guided decoupling module (SGDM) to obtain effective shallow global features, which provides favorable prior information for feature representation. Moreover, we use the cross-feature screening module (CFSM) with the dual gate control threshold strategy to enhance the interaction between feature, which further improves the feature representation. Finally, we evaluate the proposed TSDNets on three benchmark datasets. The experimental results show that our method achieves new state-of-the-art results in the classification performance with significant improvements over existing approaches.

The proposed TSDNets can extract the semantics of spatial details with high performance and efficiency. In the future, we will consider reducing the number of model parameters for a more concise and efficient spatial decoupling network. At the same time, feature screening is not sufficient, so it is necessary to further classify features according to importance: useful features, general features, redundant features.

## Data Availability

The data used to support the findings of this study are available from the corresponding author upon request.
